# Human and rat ex vivo sweat glands for the observation of acetylcholine induced intracellular calcium signalling

**DOI:** 10.1371/journal.pone.0323255

**Published:** 2025-05-08

**Authors:** Teresa Franziska Buck, Tobias Kisch, Charli Kruse, Matthias Brandenburger

**Affiliations:** 1 Institute for Medical and Marine Biotechnology, University of Lübeck, Lübeck, Germany; 2 CellTec Systems GmbH, Lübeck, Germany; 3 Department of Plastic Surgery and Hand Surgery, Praxisklinik Kronshagen, Kiel-Kronshagen, Germany; 4 Fraunhofer Research Institution for Individualized and Cell-Based Medical Engineering, Lübeck, Germany; King Faisal University, SAUDI ARABIA

## Abstract

Sweating is imperative for thermoregulatory function in humans. However, many individuals find it undesirable due to its associated malodorous nature. Despite these concerns, aluminium salts remain among the most efficacious sweat-inhibiting agents. Nevertheless, the discovery of novel antiperspirants is challenging due to restrictions on animal testing and the limitations of existing cellular models. Sweat glands, when isolated from human subjects, have been shown to possess only minor functional limitations. Consequently, they are regarded as a leading alternative, despite their limited availability. As the primary analytical metric for isolated sweat glands is sweat output, the objective of this study was to devise experiments that enhance both analysability and availability. We consequently present a method for the reliable observation of intracellular calcium responses in isolated human sweat glands ex vivo. Furthermore, we show that availability can be improved by demonstrating that isolated human sweat glands can be cryopreserved while preserving viability, structural integrity and functionality. In order to obtain these results with limited access to human sweat glands, we set up an experimental set-up using rat sweat glands and compared them to human sweat glands. The study demonstrates that isolated sweat glands from rats and humans exhibit a remarkably similar physiological calcium response to cholinergic stimulation. This validates the use of rat sweat glands as a suitable model for the development of novel experimental procedures, thus circumventing the scarcity of human sweat gland samples. This approach significantly enhanced the analysability and availability of human sweat gland samples. The calcium imaging method applied in this study facilitates the exploration of sweat gland physiology at the cellular level, thereby enabling a more detailed understanding of sweating disorders. This, in turn, enhances the suitability of the model for use in cosmetic testing and the discovery of new antiperspirant agents, thus circumventing the need for animal testing.

## Introduction

Despite the increasing utilisation of body fluids for diagnostic purposes, sweat remains a relatively underutilised resource, despite its ease of extraction from the body. In the past, the diagnostic potential of sweat has been extensively demonstrated for cystic fibrosis [1]. However, detailed insights into the physiological and pharmacological characteristics of sweating at the cellular level remain limited, primarily due to the inherent limitations of the available models and analysis methods. In order to address the remaining research questions, functional model systems have been developed, which involve test persons or access to animal tissue. Due to ethical restrictions and species limitations, especially in terms of tissue availability, the main effort has been to develop functional human cell-based sweat gland models [2,3]. Human-derived cellular sweat gland models, including the NCL-SG3 cell line, are valuable and well analysable on a cellular level. However, they lack critical features such as cholinergic responsiveness, which limits their utility for signal transduction studies [3]. While organoid-based models offer a promising level of complexity, there is uncertainty regarding their ability to fully recapitulate native gland function [2]. Ex vivo models provide an alternative, allowing direct physiological and pharmacological investigations, but mostly lack methods for analysis on the cellular level. Notwithstanding, previous studies have demonstrated the successful ex vivo stimulation of mouse sweat glands, revealing dose-dependent calcium responses to acetylcholine [4]. However, species-specific differences restrict their translational potential [5–8].

Rat sweat glands have been identified as a promising model for studying human sweat gland development and function. Following the isolation of rat paws during designated animal experiments, the glands are highly accessible and exhibit significant similarities in size [9] and developmental characteristics [10] with human sweat glands. Therefore, the present study aims to establish calcium imaging in rat and human sweat glands, with the objective of overcoming methodological limitations. Initially, rat sweat glands were utilised to establish the experimental setup, and subsequently, these procedures were transferred to human tissue samples due to the limited availability of human sweat gland samples. By staining isolated glands with calcium-sensitive fluorophores and monitoring responses using confocal laser scanning microscopy, we have demonstrated striking similarities in intracellular calcium signalling between rat and human sweat gland signalling under cholinergic stimulation in both simple and complex functional assays. Furthermore, given the limited availability of fresh human sweat gland samples, we explored cryopreservation as a method to extend tissue availability. Our findings indicate that cryopreserved human sweat glands retain viability, structural integrity, and functional responses post-thawing, even in complex experimental setups. This advancement could significantly enhance the feasibility of sweat gland research in both academic and industrial settings, offering a robust model for studying physiological signalling and pharmacological interventions.

## Materials and methods

### Sweat gland isolation

The collection of tissue for human sweat gland isolation was performed at the University Hospital Schleswig-Holstein (UKSH) following the acquisition of written informed consent from patients undergoing plastic surgical procedures. The University of Luebeck Ethics Committee had previously granted permission for the collection, storage and subsequent research (Ref: 21–111). The recruitment period commenced on 9 April 2021 and was concluded on 8 March 2022. Patients who underwent plastic surgery on the abdomen, axilla and thighs were between 25 and 61 years of age. Post-surgery, the skin was stored in 4 °C ringer solution (Fresenius, Bad Homburg, Germany) for up to one day. All steps were carried out in ringer solution to protect skin from desiccation. For the isolation procedure, approximately 20 cm^2^ of skin was selected and the subcutaneous fat was removed without damaging the dermis. The skin was then cut into pieces measuring 4 mm x 4 mm size and further minced with a rotary cutter until the pieces were undiscernible. The minced skin was then transferred into a 50 mL tube containing 20 mL of digestion media composed of Minimal Essential Medium (Sigma-Aldrich, Taufkirchen, Germany) containing 1% (w/v) Bovine Serum Albumin Fraction V (BSA, PAN Biotech, Aidenbach, Germany), 8 mM HEPES (pH 7.6, Roth, Karlsruhe, Germany), 1.44 mM glutamine (Merck, Darmstadt, Germany), 0.5 mM calcium chloride (Merck, Darmstadt, Germany) and 200 Wuensch/mL collagenase type IV (Thermo Fisher Scientific, Darmstadt, Germany) adjusted to pH 7.45–7.46. The mixture of digestion media and minced skin was then incubated at 4 °C over night. The following day, the digestion mix was purged with carbogen for 30 seconds and fixed on a 37 °C heated orbital shaker (GFL, Burgwedel, Germany) set to 150 rpm. Skin digestion was carried out for two to three hours until sweat glands were liberated from skin. Subsequently, the digestion process was terminated by at least doubling the original volume with fresh culture medium composed of Dulbecco’s Modified Eagles Medium (Thermo Fisher Scientific, Darmstadt, Germany) containing 20% fetal calf serum (FCS, Thermo Fisher Scientific, Darmstadt, Germany) and 1% penicillin-streptomycin (Merck, Darmstadt, Germany). Following the distribution of the digestion to 90 mm cell culture dishes, the isolation of sweat glands was conducted under a microscope (Axiovert 40C, Zeiss, Oberkochen, Germany) utilizing tweezers and pipettes. Thereby isolated sweat glands were transferred to fresh culture medium and cultivated at 37 °C and 5% CO_2_ until their utilisation in experiments. It is noteworthy that only eccrine sweat glands were employed in the experimental procedures undertaken in this study. An alternative source of sweat glands are curettages. In this instance, the suction material was diluted with 4 °C cold ringer solution to a total volume of at least 50 mL, mixed thoroughly and then incubated for approximately 45 min to establish phase separation. The floating fat layer was then removed and the bottom phase containing the sweat glands was harvested incrementally, diluted and single sweat glands collected as described previously.

Rat paws were obtained from male and female Wistar rats (Crl:WI and HsdCpb:WU) aged between 1.5 and 7 months after the completion of the animal experiments and subsequent donation by our animal facility and university partners. Permission was requested by our university partners and granted by the respective animal facilities. The animals were not exposed to any treatment in life known to influence sweat gland physiology. In accordance with the 3R principle, the rat paws utilised in this study were surplus material from animals sacrificed in other animal studies. The fore and hind paws were amputated above the ankle post mortem. All animal handling was performed by the university partners. For rat paws collected from partners of the University of Luebeck that had undergone experiments during their lifetime: The experimental protocols for the animals and their care in this study were in accordance with European Parliament Directive 2010/63/EU and German law and were approved by the Animal Care Committee of the State of Schleswig-Holstein, Germany. The PHS Policy on Humane Care and Use of Laboratory Animals (NIH Publication No. 15–8013, revised 2015; https://olaw.nih.gov/policies-laws/phs-policy.htm) was adhered to in all experiments. The animals were euthanised by decapitation using a guillotine, with no anaesthesia or analgesia administered. This procedure was required in the original study by our university partner. For rat paws collected from University of Lübeck partners not used in experiments during their lifetime: rats were sacrificed for organ harvesting, e.g., for teaching or other purposes. For paws collected at the University Hospital Hamburg-Eppendorf: animals were sacrificed under CO_2_ anaesthesia followed by decapitation. All animal experiments were performed in accordance with the ARRIVE guidelines. All animal experiments were approved by the Ministry of Justice and Consumer Protection and the Free and Hanseatic City of Hamburg and conducted in accordance with the guidelines of the Animal Protection Committee of the University Medical Center Hamburg (Hamburg, Germany) under approval numbers Org766 and Org1088.

Isolation of sweat glands was carried out analogously to the procedure described for human skin samples.

### Cryopreservation and thawing of isolated human sweat glands

In order to enhance availability, isolated human sweat glands were subjected to a process of cryopreservation and stored in the gas phase of liquid nitrogen at approximately -155 °C. For this purpose, five sweat glands were transferred together in a cryo tube with 500 µ L of cryopreservation media consisting of 10% dimethyl sulfoxide (DMSO, Roth, Karlsruhe, Germany) and 90% FCS. The tube was then cooled at a rate of -1 °C/min to -80 °C, after which it was transferred to a liquid nitrogen tank for long-term storage.

The cryopreserved sweat glands were thawed by removing a cryovial with sweat glands from the liquid nitrogen storage and warming it at 37 °C in water quench until only a small piece of ice was visible. Thereafter, 500 µ L of pre-warmed culture media was added, and the suspension was carefully transferred to a round culture dish containing 10 mL of fresh media. Sweat glands were then cultured at 37 °C, 5% CO_2_ for a minimum of two and a half hours prior to utilisation in downstream experiments.

### Immobilization of sweat glands on surfaces

In order to prevent delocalization of the sweat glands during the course of the experiments, the sweat glands were immobilized with either 6 µg/cm^2^ CellTak™ (Corning, NYC, New York) or MAPcell (Sigma-Aldrich, Taufkirchen, Germany). The culture dishes were coated in accordance with the suppliers’ adsorption protocol. For the immobilization of sweat gland, the desired number of glands in combination with the designated buffer or culture medium were applied to the coated surfaces. The buffer was then removed with care to allow the sweat glands settle at their intended locations. Following a four-minute incubation period without buffer at room temperature, the sweat glands attached to the surface. The buffer was then added, and the immobilised sweat glands were used for subsequent experiments.

### Live-dead staining of isolated sweat glands

The vitality of sweat glands can be microscopically evaluated via live-dead-staining, which is based on metabolic activity of vital cells and increased membrane permeability of dead or damaged cells for discrimination. To this end, the sweat glands were subjected to staining with 8 µg/mL fluorescein-diacetate (FDA, Sigma-Aldrich, Taufkirchen, Germany), 20 µg/mL propidium iodide (PI, Sigma-Aldrich, Taufkirchen, Germany) and 20 µg/mL Hoechst33342 (Thermo Fisher Scientific, Darmstadt, Germany) for a duration of 15–30 minutes. The staining buffer comprised 115 mM NaCl (Roth, Karlsruhe, Germany), 5.4 mM KCl (Roth, Karlsruhe, Germany), 1 mM MgCl_2_ (Roth, Karlsruhe, Germany), 2 mM CaCl_2_ (Merck, Darmstadt, Germany), 20 mM HEPES (Roth, Karlsruhe, Germany) and 10 mM glucose (Roth, Karlsruhe, Germany). The pH was adjusted to pH 7.42 at 37 °C.

Three-dimensional stacks in z-axis were recorded at the confocal laser scanning microscope 710 (LSM, Zeiss, Oberkochen, Germany) using ZEN black imaging software (Version 2.1 SP3). Subsequently, a maximal intensity projection was created.

### Indirect immunofluorescence

#### Staining of human full-thickness skin sections.

For indirect immunofluorescence staining of human full-thickness skin sections containing sweat glands, fresh human skin samples were first embedded in Tissue-Tek® O.C.T.™ and then divided into 12 µm sections, which were stored at -20 °C.

For the staining of the microscope slides containing cryo sections, thawing at room temperature for 30 minutes was conducted, followed by fixation for ten minutes with 4% paraformaldehyde (PFA, Merck, Darmstadt, Germany) and 10% sucrose (Merck, Darmstadt, Germany). Subsequently, the skin sections were washed three times with Dulbecco’s phosphate buffered saline solution (DPBS, Thermo Fisher Scientific, Darmstadt, Germany) and permeation was performed with 0.1% Triton X-100 (Fluka, Hannover, Germany) in DPBS. Following this step, 10% normal goat serum (NGS, Vector Laboratories, Newark, California, United States) diluted in DPBS was applied for a minimum of 30 minutes to block unspecific antibody binding. The respective primary antibodies employed included anti-CHRM3 (rabbit polyclonal, 0.03 µg/mL, HPA048036, Sigma Aldrich, Taufkirchen, Germany, RRID: AB_2680238), anti-αSMA (mouse monoclonal, 0.44 µg/mL, M0851, Dako, Düsseldorf, Germany, RRID: AB_2223500), anti-Cx26 (mouse monoclonal, 5 µg/mL, 13–8100, Thermo Fisher Scientific, Darmstadt, Germany, RRID: AB_2533036) or anti-CK7 (rabbit polyclonal, 4 µg/mL, ab53123, Abcam, Cambridge, United Kingdom, RRID: AB_869896) and appropriate IgG controls were diluted in Tris-buffered saline containing 10 mM Tris (Roth, Karlsruhe, Germany), 150 mM NaCl, 0.05% Triton X-100 and 0.1% BSA and cryo sections were incubated over night at 4 °C.

The following day, the skin sections were washed with DPBS and then incubated for one hour at 37 °C with the specific secondary antibodies, namely Cy3-anti-mouse (115-165-166, Dianova, Hamburg, Germany, RRID: AB_2338692), FITC-anti-rabbit (111-095-144, Dianova, Hamburg, Germany, RRID: AB_2337978) or FITC-anti-chicken (103-095-155, Dianova, Hamburg, Germany, RRID: AB_2337384) in DPBS. Skin sections were then washed with DPBS, and nuclei were stained with 1 µg/mL DAPI (Roche, Basel, Switzerland) in DPBS for ten minutes. Following a wash in DPBS twice and purified water the skin sections were covered with vectashield (Vector Laboratories, Newark, California, United States) and a cover slip. Microscopy was performed at the fluorescence microscope Zeiss Observer Z1 (Zeiss, Oberkochen, Germany).

#### Whole-mount staining of isolated sweat glands.

For the immunofluorescence staining of isolated sweat glands, the glands were initially immobilised in an ibidi culture dish (ibidi, Gräfelfing, Germany) in accordance with the previously described protocol. For the purpose of fixation, either 4% PFA at room temperature or a mixture of methanol (Roth, Karlsruhe, Germany) and acetone (Roth, Karlsruhe, Germany) at a ratio of seven to three at -20 °C was utilised. All subsequent incubation steps were carried out for 48–72 hours at 4 °C, unless stated otherwise. Following fixation, the sweat glands were washed for a minimum of five minutes with a washing buffer consisting of DPBS with 0.1% Triton X-100 and 0.1% Tween-20 (VWR, Radnor, Pennsylvania, United States). Primary antibodies were then added in whole-mount blocking buffer consisting of wash buffer with 2% BSA added. After washing out residual primary antibody using washing buffer three times for at least five minutes, the secondary antibodies and DAPI were diluted in whole-mount blocking buffer and incubated. Sweat glands were then again washed three times for five minutes. After that, sweat glands were cleared of residual pigmentation using Scale S4 clearing solution [11]. Finally, the clearing solution was removed and the sweat glands were covered with vectashield and a round glass cover slip (Roth, Karlsruhe, Germany) before three-dimensional imaging at the confocal laser scanning microscope LSM 710.

### Calcium imaging

In order to observe intracellular calcium ion signal transduction, immobilized sweat glands were stained with calcium-sensitive fluorescent dyes with a protocol adapted from Klar et al. (2014). The immobilized sweat glands were loaded with 10 µ M Fura Red™-AM and Fluo-4-AM each, supported by adding 0.04% (v/v) Pluronic F-127 (Biomol, Hamburg, Germany) in buffer containing 115 mM NaCl, 5.4 mM KCl, 1 mM MgCl_2_, 2 mM CaCl_2_, 20 mM HEPES, 10 mM glucose and 25 mg/mL BSA adjusted to pH 7.42 at 37 °C. Following an overnight incubation of the sweat glands at 28 °C without gassing, the buffer was replaced and the sweat glands incubated for a duration of 30 minutes at the same temperature. Thereafter, the immobilized sweat glands were subjected to different stimulation protocols.

The measurements were conducted at a temperature of 37 °C using a confocal laser scanning microscope (LSM 710) with ZEN black imaging software (Version 2.1 SP3). Initially, a three-dimensional image recording in the z axis was performed for the generation of a maximal intensity projection prior to stimulation. Subsequent to this, a time series was initiated, commencing with the recording of resting calcium levels for minimum of two minutes. Thereafter, the stimulation of sweat glands was conducted using the designated agonist, followed by the administration of 3 µ M ionomycin as a technical control. Ionomycin facilitates the unobstructed influx of Ca^2+^ into cells, irrespective of physiological relevant stimulation and thereby demonstrating the maximal colour change and adequate dye loading. Fluorescence was recorded by exciting both dyes at 488 nm and setting the emission filters for Fura Red™ to 633–758 nm and for Fluo-4 to 500–550 nm. Images were captured at an interval of five seconds. Subsequent to the completion of the times series recording, an additional image recording in the z axis was conducted for the purpose of generating a maximal intensity projection.

The double staining of sweat glands with Fura Red™ and Fluo-4 creates a double requirement for an intracellular calcium concentration increase in which the Fura Red™ fluorescence must decrease while Fluo-4 increases at the same time. Only when these two prerequisites are fulfilled it is reasonable to calculate the calcium response that increases when the intracellular calcium concentration increases.

### Statistical analysis

For the purpose of calcium imaging analysis, a region of interest was defined, covering the sweat gland, in order to extract fluorescence raw data for Fura Red™ and Fluo-4. Initially, the fluorescence raw data was adjusted for the specific detection gain used (all graphs depict corrected values). Then, the ratio of Fura Red™ and Fluo-4 was calculated and the mean of the first ten cycles was normalized to 100%. Subsequently, to retrieve the calcium responses, the individual values were then subtracted from 100%. For the construction of column graphs, the values for specific stimuli were retrieved by averaging ten values after the maximal calcium response and then calculating the mean and SEM of independent experiments (GraphPad PRISM 5, standard error of the mean).

Data is expressed as the mean ± SEM. The statistical significance of the differences between the means of the data from two groups were determined by a two-tailed Student’s t-test with an α level of 0.05. E.g. calcium response of 1 µ M mACh stimulated vs. unstimulated or freshly isolated vs. cryopreserved sweat glands were compared. P-values < 0.05 were considered significant. Statistical significance was determined using GraphPad PRISM statistical environment, version 5.04.

## Results and discussion

### Calcium imaging reveals functional similarities between rat and human sweat glands in cholinergic stimulation

The rat and human sweat gland cholinergic calcium responses imaged by calcium imaging were found to be composed of two characteristics: firstly, an increase in intracellular [Ca^2+^] was visible by a colour switch from red (Fura Red™) to green (Fluo-4); and secondly, a secretory coil contraction.

During baseline recording in the absence of stimulation no change in fluorescence intensity was observed for either Fura Red™ or Fluo-4 in either rat or human sweat glands. However, upon the administration of 1 µ M acetyl-β-methylcholine (mACh), a marked decline in Fura Red™ fluorescence was observed, concomitant with an increase in Fluo-4 fluorescence manifested in both species. This finding indicates that the dual requirement for an increase in intracellular calcium concentration was met. As this reaction is in accordance with the Gα_q_ coupled receptor signalling of CHRM3 receptor that was stimulated [12,13], it was reasonable to calculate the calcium response for both species as described in the Materials and Methods section. The calcium responses of rat sweat glands exhibited a plateau at 1 µ M mACh concentrations, reaching an average calcium response of 47.4 ± 4.0% for 1 µ M mACh (n = 4). The calcium response of 10 µ M mACh was only slightly lower with an average of 40.8 ± 4.7% (n = 4). However, the subsequent influx of calcium into the cells, facilitated by ionomycin as a technical control, resulted in a pronounced colour change, with an average intensity of 77.0 ± 4.0% (n = 4), as evident in the representative fluorescence images. The variability of individual calcium responses of different sweat glands was almost 20% in rats (n = 4).

**Fig 1:**
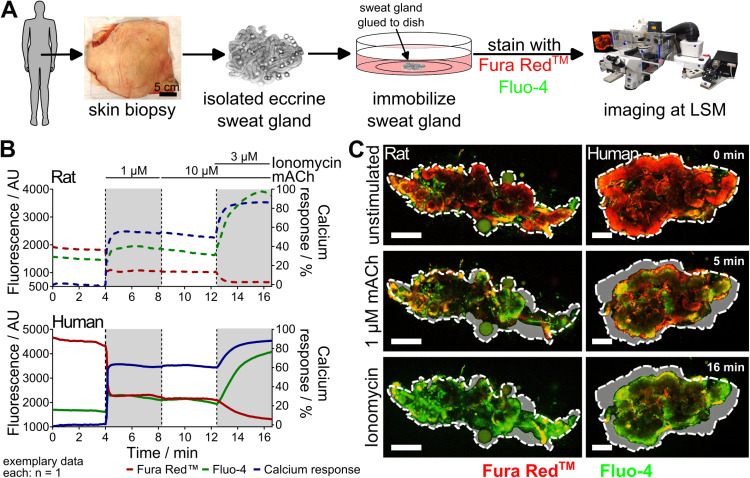
Calcium imaging reveals comparable calcium responses and contractions in rat and human sweat glands. (A) Calcium imaging workflow: isolation of rat and human sweat glands via enzymatic digestion, immobilization, and calcium dye staining for imaging. (B) Cholinergic stimulation of sweat glands with mACh induces a sharp Fura Red™ (red) decrease and Fluo-4 (green) increase in both species, indicating a strong intracellular calcium rise (exemplary depiction). Ionomycin serves as a positive control. The corresponding calcium response is shown in blue. (C) Representative fluorescent images show the Fura Red™ to Fluo-4 colour switch and secretory coil contraction upon mACh stimulation. Dashed line: gland contour before stimulation. Light grey area: contraction difference. Acetyl-β-methylcholine (mACh), arbitrary units (AU), laser scanning microscope (LSM), Scale bars: 100 µm.

As well, it has been demonstrated that in the case of human sweat glands, the calcium response in all replicates reached a state of saturation at a concentration of 1 µ M mACh with an average value of 54.0 ± 7.7% (n = 3). An increase in concentration to 10 µ M mACh did not result in a significant enhancement of the effect, and over time, a modest decline to 52.1 ± 9.0% was observed (n = 3). However, the addition of ionomycin as a technical control resulted in a significant enhancement of the calcium response, reaching 83.8 ± 1.6% (n = 3). The variability in the calcium response among individual human sweat glands was approximately 30% (exemplary data in Fig 1B).

A detailed examination of fluorescence images over time revealed that the colour changes were restricted to the inner part of the tubules in human sweat glands. This restriction was not as clearly evident in the rat sweat glands, presumably due to their comparatively smaller size.

In addition to the calcium response, a secretory coil contraction was observed in both rat and human sweat glands that was both persistent and not enhanced by higher mACh concentrations than 1 µ M (Fig 1C).

As demonstrated by sweating experiments, both isolated rat and human sweat glands respond to the stimulation by cholinergic agents with a persistent sweat secretion over several hours [5,14,15]. To achieve this, 1 µ M methacholine as an equivalent stimulus that agonises muscarinic acetylcholine receptor M3 (CHRM3) was used. Furthermore, Salpeter and Eldefrawi calculated the acetylcholine concentration in the synaptic cleft of rodents to be 10 µ M [16]. Therefore we conclude that the mACh concentrations of 1 µ M and 10 µ M used in our publication are appropriate for the cholinergic stimulation. The constant calcium response could be indicative for the long-lasting and constant sweat secretion observed in ex vivo sweat glands [5,15]. The relatively minor decline in calcium response during the experiment could be indicative of a decrease in intracellular calcium concentration through transport into the extracellular space or back into the endoplasmatic reticulum [17]. However, this decline could also be attributed to unequal dye bleaching [18] or sweat gland exhaustion due to the time-consuming staining procedure, which may have affected the smaller more fragile rat sweat glands to a greater extent than the larger human sweat glands. The variability in size of rat and human sweat glands may have contributed to the differences in calcium response variability, which was 20% in rats and 30% in humans. While rat sweat glands, with an average diameter of approximately 400 µm [5], are smaller and more consistent in size, human sweat glands display a large size variation, ranging from 500 µm to 700 µm or larger, depending on individual and regional skin variations [6,7].

The second characteristic of the cholinergic calcium response that rat and human sweat glands share is the contraction. While there is no report on this phenomenon in rat sweat glands [5], it is a clearly reproducible characteristic of both species. Further analysis of the images captured during stimulation provides a more detailed insight into the location of the calcium response, particularly evident in human sweat glands. Specifically, the inner secretory coil cells exhibit a colour change of red to green, indicative of a substantial intracellular increase in [Ca^2+^] [8,10,19,20], while the surrounding cells, presumed to be myoepithelial cells involved in the secretory coil contraction, remain largely unchanged [21–23]. This observation suggests the locally or temporally regulated calcium response in these cells [24], thereby unveiling hitherto unreported cellular intricacies.

Despite the evident parallels in the stimulation of cholinergic receptors, a significant divergence emerges when considering the functional implications of rat and human sweating. While human eccrine sweat glands can also be stimulated by α_1_-adrenergic [9,14,25] and β-adrenergic stimulants [26,27], rat sweat glands, in contrast, cannot be stimulated by β-adrenergic agonists [28,29]. Furthermore, studies on α_1_-adrenergic stimulation are still ambiguous [28–31]. This hinders the utilization of rat sweat glands as a human model systems. Furthermore, human eccrine sweat glands are found almost all over the body and their main function is body temperature regulation in hot climates [32]. In contrast, rat sweat glands are found exclusively beneath the paws and function to increase friction, with rats using their tails for thermoregulation [8,33]. Despite these limitations, rat sweat glands from rat paws are very easy to access because in many animal experiments the paws remain untreated and therefore after completion of animal experiments, those can be used. In 2020, a total of 132,832 rats were euthanised in Germany [34]. Compared to approximately 23,000 relevant human plastic surgeries [35] this is approximately six times the number of samples potentially available for human sweat gland isolation. When considering the potentially greater availability of rat sweat glands compared to human sweat glands and the pharmacological differences between the species, the utilization of rat sweat glands could be favourable for the establishment of experiments using cholinergic stimulation and for the saving of human samples. However, for detailed pharmacological investigations relevant to humans, the use of human sweat glands remains essential.

### Cryopreserved human sweat glands retain viability, structural integrity and functionality

In order to enhance the efficiency of human sweat glands in experimental contexts and to mitigate reliance on human sweat glands from fresh skin samples, which have a limited shelf-life of a few hours to a few days post-isolation [36], isolated human sweat glands were cryopreserved in accordance with the material and methods section. Following thawing, viability, structural integrity and functionality were assessed.

**Fig 2:**
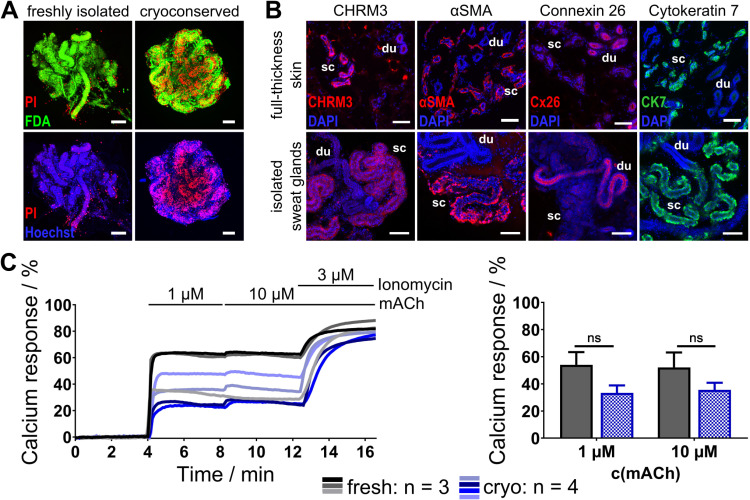
Cryopreserved human sweat glands maintain vitality, structure and responsiveness to cholinergic stimulation. (A) In comparison to freshly isolated glands, cryopreserved glands show an increased number of PI-positive nuclei (cell damage) but retain FDA staining, indicating metabolic activity post-thaw after cultivation for several hours. (B) Furthermore, immunofluorescence confirms the preservation of key structural and functional markers in whole-mount stained cryopreserved sweat glands when compared to native full-thickness skin sections. (C) Furthermore, calcium imaging demonstrates cholinergic responsiveness of thawed sweat glands (n = 4), with no significant difference observed when compared to fresh sweat glands (n = 3) (p = 0.0980 for 1 µ M mACh, p = 0.1967 for 10 µ M mACh). Acetyl-β-methylcholine (mACh), arbitrary units (AU), α smooth muscle actin (αSMA), acetylcholine receptor M3 (CHRM3), cytokeratin 7 (CK7), connexin 26 (Cx26), DAPI/Hoechst (nuclei stain), duct (du), fluorescein diacetate (FDA), propidium iodide (PI), secretory coil (sc). Data represents mean ± SEM. ns: p ≥ 0.05, Two-tailed Student’s t-test. Scale bars: 100 µm.

Initially, the viability of freshly isolated and thawed human sweat glands was examined using live-dead staining, as this is the primary criterion for assessing their functional conservation. Freshly isolated sweat glands exhibited a strong FDA-positive staining, indicating that cells within these glands were metabolically active and vital. Conversely, PI-positive stained cell nuclei were barely visible, with the exception of the ducts where the sweat glands had been dislocated from the skin. Overall, the sweat glands appear to be largely undamaged after the isolation process. In contrast, cryopreserved and thawed sweat glands exhibited elevated numbers of PI-positive nuclei, dispersed throughout the glandular structure. However, these glands retained significant FDA-positive staining, suggesting that metabolic activity was preserved post-thawing. This observation indicates that not all sweat gland cells were fatally damaged (see Fig 2A). In summary, following thawing, the cells exhibited a significant degree of green FDA-positive staining, concurrently demonstrating numerous PI-positive cells. This dual staining could be attributed to the composition of the cryopreservation medium, which contains DMSO as a permeating cryoprotectant, which would render cell membranes permeable to PI for a limited time after thawing until the membranes have recovered. However, no specific cell types or regions seem to be exclusively damaged, as PI-positive cells were observed throughout the sweat glands. This is encouraging, given that sweat glands are composed of at least four different cell types, and each cell type probably has its own optimal freezing and thawing conditions, which makes cryopreservation of multicellular objects exceptional challenging [37].

In light of the finding that cryopreserved and thawed sweat glands retained partial viability, the structural integrity of the sweat glands was assessed via indirect immunofluorescent staining of of key proteins in sweating. To this end, a comparison was made between cryopreserved and thawed isolated human sweat glands and full-thickness skin sections, which served as a positive control for the observed staining patterns. As we had previously demonstrated cholinergic stimulation via CHRM3 receptor in fresh human samples in the established calcium imaging assay, we checked for CHRM3 staining. In the context of skin sections, there was a pronounced unstructured staining of the secretory tubules, but this was absent in the duct. This staining was also evident in the thawed sweat glands, with the secretory coil being the only positive staining, while the duct was negative for CHRM3. This suggests that CHRM3 is conserved among skin sections and thawed sweat glands. We selected αSMA as an indicator for structural integrity and function, given that only myoepithelial cells surrounding the secretory coil express this protein. The function of myoepithelial cells is to support secretory coil contractions to facilitate sweat output. In skin sections, αSMA-positive stained cells were only visible around secretory coils where myoepithelial cells are located, while the inner secretory coil part and ductal regions remained unstained. In the context of thawed sweat glands, optical sections revealed a distinct location of positive staining at the periphery of the secretory tubules, while the ductal and inner regions of the secretory tubules remained negative for αSMA, thereby confirming the preservation of αSMA post-thawing. During the process of production and transportation of sweat to the skin’s surface, a process of reabsorption of water occurs within the duct. To do this, ductal cells form a syncytium by connecting with each other through gap junctions. Connexin 26 (Cx26) is a protein that is characteristic of ductal cells and is essential for the establishment of these tightly regulated cell-cell connections. In full-thickness skin sections only the inner parts of the ducts were stained Cx26 positive, while the secretory tubules were negative. In thawed sweat glands even the membranous location of Cx26 in the duct became clearly visible while the secretory coil was unstained. Consequently, the staining pattern of this protein remained unchanged after thawing. Finally, it is important to note that cytokeratins mediate mechanical stability in cells. Cytokeratin 7 (CK7) is expressed specifically in the secretory region of sweat glands, while the duct is negative for CK7. This staining pattern was evident in the full-thickness skin sections and, moreover, in the thawed sweat glands. In both images, the positive stained secretory regions exhibited an extensive staining pattern, indicating a structural conservation of the protein (see Fig 2B).

In conclusion, a comparison of native human full-thickness skin sections with cryopreserved and thawed sweat glands revealed no significant differences in the staining patterns of the functional or structural relevant sweat gland proteins we investigated. These results complement the broad metabolic activity indicated by the overall FDA-positive staining, providing an additional indication that thawed sweat glands could retain functionality.

In order to provide the final proof regarding the functionality of cryopreserved sweat glands, thawed sweat glands were used in calcium imaging experiments, with no alterations being made to the experimental procedures. The application of 1 µ M mACh resulted in a substantial increase in Fluo-4 fluorescence, concurrently with a decrease in Fura Red™ fluorescence. This led to a substantial calcium response that was reasonable to calculate as all requirements were met. The administration of 10 µ M mACh resulted in a further modest increase in calcium response in all examined thawed sweat glands. The average calcium responses elicited by 1 µ M and 10 µ M mACh were recorded as 33.3 ± 4.8% and 35.6 ± 4.5% (n = 4), respectively. The variability observed among the examined sweat glands was approximately 25%, which was slightly lower than that seen for freshly isolated sweat glands. While calcium responses of cryopreserved sweat glands exhibited a tendency to be lower, these values did not differ significantly from those observed in freshly isolated sweat glands (p = 0.0980 for 1 µ M mACh and p = 0.1967 for 10 µ M mACh) (see Fig 2C). Furthermore, thawed sweat glands also exhibited secretory coil contractions that were slightly less intense than those observed in freshly isolated sweat glands (S1 Fig). The reduction in calcium response and contraction could be a result of cell damage through intracellular ice crystal formation during freezing and thawing [38,39]. Optimization strategies may encompass pre-incubation steps of sweat glands in freezing medium [40], the addition of other permeating or non-permeating cryoprotectants to the actual medium [41–45], or the optimization of freezing and thawing speed [46]. Notwithstanding, it was demonstrated that thawed sweat glands retain responsiveness to cholinergic stimulation at concentrations that are relevant to physiological processes. In view of the results obtained, it was decided to include the results of thawed human sweat glands in subsequent experiments and analysis.

As demonstrated by this study, isolated human sweat glands can be successfully cryopreserved while maintaining viability, structural integrity, and functional responsiveness. The findings of the present study serve to enhance the efficiency of the utilisation of isolated human sweat glands, whilst concomitantly effecting as substantial reduction in dependency on fresh skin samples. The applicability of human sweat glands in test systems is enhanced, due to the extension of storage and temporal availability is extended from several post-isolation to potentially years or even decades. This technique possesses the potential to improve the model availability thereby facilitating the utilization in cosmetic and pharmacological testing, thus providing an alternative to animal studies.

### Rat and human sweat glands exhibit similar behavior in functional calcium imaging, even in complex experimental setups

In order to test the pharmacological responsiveness of sweat glands to specific agonists and their complementing antagonists, it was necessary to assess whether isolated sweat glands could be repeatedly stimulated in the fluorescent calcium imaging assay. Again, rat sweat glands were used for the initial setup of experimental procedures. These results were then transferred to human samples.

**Fig 3:**
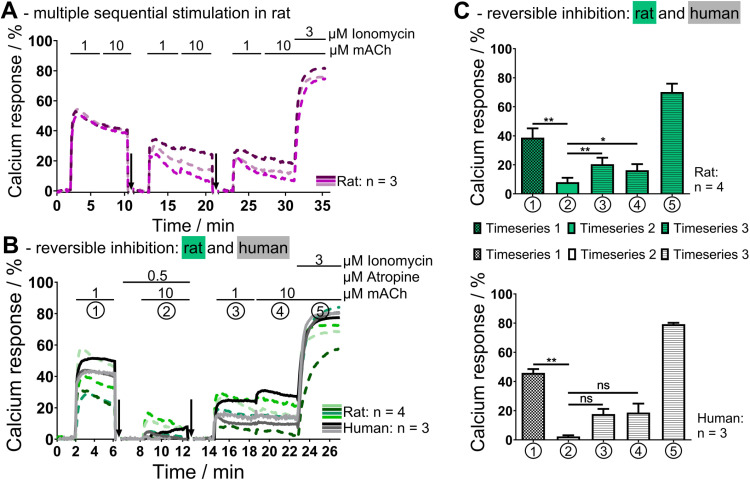
Rat and human sweat glands tolerate multiple sequential stimulations for pharmacological studies. (A) Rat sweat glands were used to assess repeated stimulations of the same gland, revealing a gradual decline in maximal calcium response (n = 3). (B) These findings guided the evaluation of atropine’s reversible inhibition in rat (dashed green lines, n = 4) and human (solid grey lines, n = 3) sweat glands. (C) In rat sweat glands, atropine significantly inhibited cholinergic stimulation (p = 0.0087, timeseries 1 vs. 2), with full reversibility after washout (p = 0.0067 for 1 µ M, p = 0.0171 for 10 µ M mACh, timeseries 2 vs. 3). In human sweat glands, the inhibition was found to be significant (p = 0.0037, timeseries 1 vs. 2), however, the reversal was not (p = 0.0544 for 1 µ M, p = 0.1239 for 10 µ M mACh), although the trends did correspond to the rat sweat gland data. Downward arrows indicate the wash and regeneration steps. Acetyl-β-methylcholine (mACh). Data represent mean ± SEM. ns p ≥ 0.05, * p < 0.05, ** p < 0.01, Student’s t-test.

The first step was to test, whether rat sweat glands could be repeatedly stimulated with the same cholinergic stimuli. This was indeed the case, as the initial response to 1 µ M mACh was 51.8 ± 0.8%. After a wash and recovery step, the calcium response for the second stimulation was 29.0 ± 2.3% and finally 23.3 ± 1.6% for the third stimulation (n = 3). The re-stimulated sweat glands, after each regeneration period, showed all the characteristics of their calcium response described previously in this publication, namely a sharp increase in intracellular calcium concentration and a contraction of the secretory coil. The final calcium response during the third round of stimulation was reduced but still 45% of that during the first round of stimulation (see Fig 3A). Reasons for the observed reduction could be a result of sweat gland exhaustion or fluorescent dye depletion as described above, or an artefact of isolation. Furthermore, it could be speculated that the higher calcium response during the first stimulation could be due to the effect of denervation hypersensitivity with a post-stimulus endocytotic desensitisation [47–49]. In the absence of a neuronal component after isolation, either membrane receptor expression could have increased or the membrane potential could have changed, both leading to membrane hypersensitivity [50]. Together with a increased receptor turnover and endocytotic activity, this could have lead to a rapid desensitisation of the membrane within experimentally relevant timescales [51,52]. As an appropriate control, the reduction of the calcium response over the multiple stimulation series helps to guide the evaluation of the following experiments.

In the subsequent stage of the study, the focus shifted to the evaluation of multiple stimulations of a single sweat gland in a pharmacologically relevant experiment. This was achieved by assaying the the effect of atropine inhibition on the cholinergic calcium response in rat and human sweat glands. The experimental setup consisted of an initial cholinergic stimulation, followed by a stimulation during reversible inhibition with atropine, and a final stimulation with mACh after atropine washout. As anticipated, the results obtained form the rat sweat glands demonstrated a reversible and significant effect of atropine on the cellular calcium response. The respective calcium responses were 38.7 ± 5.5% for 1 µ M mACh without atropine inhibition, followed by 8.1 ± 2.6% for 10 µ M mACh during atropine inhibition and finally 20.4 ± 3.9% for 1 µ M mACh and 16.2 ± 3.7% for 10 µ M mACh after atropine washout during the third stimulation (each n = 4). Consequently, the calcium response elicited by the third stimulation was approximately 50% of the initial calcium response, as previously observed for multiple sequential stimulations of rat sweat glands without the addition of inhibitory substances. Furthermore, the absence of secretory coil contraction during atropine inhibition confirmed the successful inhibitory effect on rat sweat glands, including all aforementioned characteristics observable in the calcium imaging assay (S2 Fig). Statistical analysis revealed a significant reduction with p = 0.0087 between the initial stimulation without atropine and the second stimulation during atropine inhibition. The reversibility of stimulation was as well significant, with p = 0.0067 and p = 0.0171 when comparing calcium responses during atropine inhibition with 1 µ M and 10 µ M mACh stimulations after atropine washout, respectively.

As the results were highly reproducible, the next step was to apply reversible cholinergic inhibition to human sweat glands. The initial calcium response to 1 µ M mACh was 45.9 ± 2.1% in human sweat glands, while the second calcium response during atropine inhibition was only 2.3 ± 0.6% for 10 µ M mACh. This difference was found to be statistically significant with a p-value of 0.0037. Following the removal of atropine, all human sweat glands exhibited a renewed responsiveness to 1 µ M and 10 µ M mACh, manifesting in elevated calcium responses of 17.7 ± 2.9% and 18.7 ± 5.0%, respectively (each n = 3). Although the values of the second stimulation during atropine inhibition did not demonstrate a significant difference compared to the third stimulation after atropine washout (p = 0.0544 for 1 µ M and p = 0.1239 for 10 µ M mACh), a clear tendency towards restimulation of human sweat glands after atropine washout was observed. This observation is particularly noteworthy when considering the experimental results of multiple sequential stimulations of rat sweat glands where the calcium response of the third stimulation was 45% of the first stimulation calcium response. Here, the calcium response to 1 µ M mACh of the third round was approximately 40% of the initial calcium response, which is only 5% lower than seen for the third stimulation in rat sweat glands (Fig 3B and C ). Furthermore, contractions of the secretory coil were only observed in the absence of inhibition. It is noteworthy, that among the human sweat glands examined was also one cryopreserved sweat gland that was effectively inhibited during atropine inhibition and could be restimulated in the third stimulation round. The presence of a calcium response supports the findings that human sweat glands can be cryopreserved using the method outlined in this publication. Furthermore, the functionality of stored human sweat glands is not only applicable for simple but also for complex experiments. Nevertheless, these results should be confirmed using a larger batch of cryopreserved sweat glands to ascertain whether the results are similar to those observed in simple experiments.

## Conclusion

The utilisation of rat sweat glands as a reliable model for human sweat glands has been demonstrated to reduce the necessity for rare human samples, given their analogous responses to pertinent stimuli. In comparison to the NCL-SG3 sweat gland cell line, which exhibits a lack of cholinergic and α_1_-adrenergic responsiveness but is stimulable by β-adrenergic stimulation [3], overall rat sweat glands demonstrate a greater degree of functional similarity with human glands (see Table 1). This renders them a more advanced model for establishing experimental setups.

**Table 1:** Comparison of pharmacological relevance of rat eccrine sweat glands, human sweat glands and human sweat gland cell line NCL-SG3.

	Rat sweat glands	Human sweat glands	Human NCL-SG3
Cholinergic stimulation	Y	Y	(no)
Cholinergic inhibition	Y	Y	NA
ɑ_1_-adrenergic stimulation	(Unclear)	(Y)	(no)
β-adrenergic stimulation	(no)	(Y)	(Y)
Contraction	Y	Y	NA

Table includes the major human sweat gland stimuli and contractability as a major physiological feature. Information in brackets from [3,5,14,15,28–31].

Furthermore, it has been demonstrated that cryopreserved human sweat glands retain viability, structural integrity, and functionality, thereby extending their usability from hours or days to weeks, months, or potentially years post-isolation. Furthermore, the capacity of these glands to undergo repeated stimulations lends further support to their application in research.

This ex vivo sweat gland model, when combined with functional imaging techniques, enables researchers to explore intracellular signalling in detail and correlate findings with actual sweat secretion. This model holds considerable promise in advancing our understanding of sweat gland physiology, improving experimental approaches for sweat-related disorders, and serving as a platform for safety screenings of pharmaceutical and cosmetic compounds. Furthermore, it may support the development of novel treatment strategies for conditions affecting sweat gland function, such as hyperhidrosis or anhidrosis, by providing insights into molecular mechanisms and potential therapeutic targets.

## Supporting information

S1 Fig Comparison of contraction of human sweat glands in calcium imaging directly after isolation and after cryopreservation. Freshly isolated and cryopreserved sweat glands differed in the intensity of contraction to cholinergic stimulus during calcium imaging. The freshly isolated sweat glands showed a greater contraction to 1 µ M mACh than the cryopreserved sweat glands. Although the contraction of the cryopreserved sweat glands was weaker than that of freshly isolated sweat glands, the contraction was clearly visible. Dotted line: outline of the sweat glands at the at the beginning of the experiment. Area highlighted in light grey: difference between the outline of the sweat gland before contraction at the time shown in the maximum projection. Acetyl-β-methylcholine (mACh), scale bars: 100 μm.
(TIF)

S2 Fig Contraction of secretory coils in response to cholinergic stimulation with and without atropine inhibition during calcium imaging of rat sweat glands. Representative images of a cholinergically stimulated rat sweat gland at different time points of the experiment. Timeseries 1 without atropine inhibition shows a clear and strong contraction of the secretory coil as well as a calcium response indicated by the colour change from Fura Red™ (red) to Fluo-4 (green). After mACh washout and application of atropine during the regeneration phase, the sweat gland is relaxed at the beginning and restimulation fails during ongoing atropine inhibition in timeseries 2. No contraction or colour change is seen. This indicates an effictive competitive inhibition of cholinergic calcium signalling by atropine. Atropine was then washed out and timeseries 3 followed the regeneration phase. Once again, the sweat gland was stimulated by cholinergic stimulation and showed a clear contraction and change in colour. This response was less pronounced than in timeseries 1. However, it clearly demonstrates the reversibility of atropine inhibition. The technical control of ionomycin results in a maximum colour change independent of physiological stimulation (positive control). Dotted line: outline of the sweat glands at the at the beginning of the experiment. Area highlighted in light grey: difference between the outline of the sweat gland before contraction at the time shown in the maximum projection. Acetyl-β-methylcholine (mACh), scale bars: 100 μm.
(TIF)

S3 File Calcium imaging raw data of Fura Red™ and Fluo-4 for graphs Fig. 1–3 and exemplary calculation of calcium response. The initial worksheet comprises raw data for Figure 1, in addition to a sample calculation of the calcium response for a cholinergically stimulated rat sweat gland. The second worksheet contains raw data from freshly isolated and cryopreserved human sweat glands during the cholinergic stimulation shown in Figure 2. The third worksheet contains raw data for multiple sequential cholinergic stimulation of rat and human sweat glands with and without atropine inhibition, as shown in Figure 3.
(XLSX)
