# Supplementary figures and images for: Human and rat ex vivo sweat glands for the observation of acetylcholine induced intracellular calcium signalling
Source: PLoS One. 2025 May 8;20(5):e0323255. doi: 10.1371/journal.pone.0323255 (PMC12061121; doi:10.1371/journal.pone.0323255)

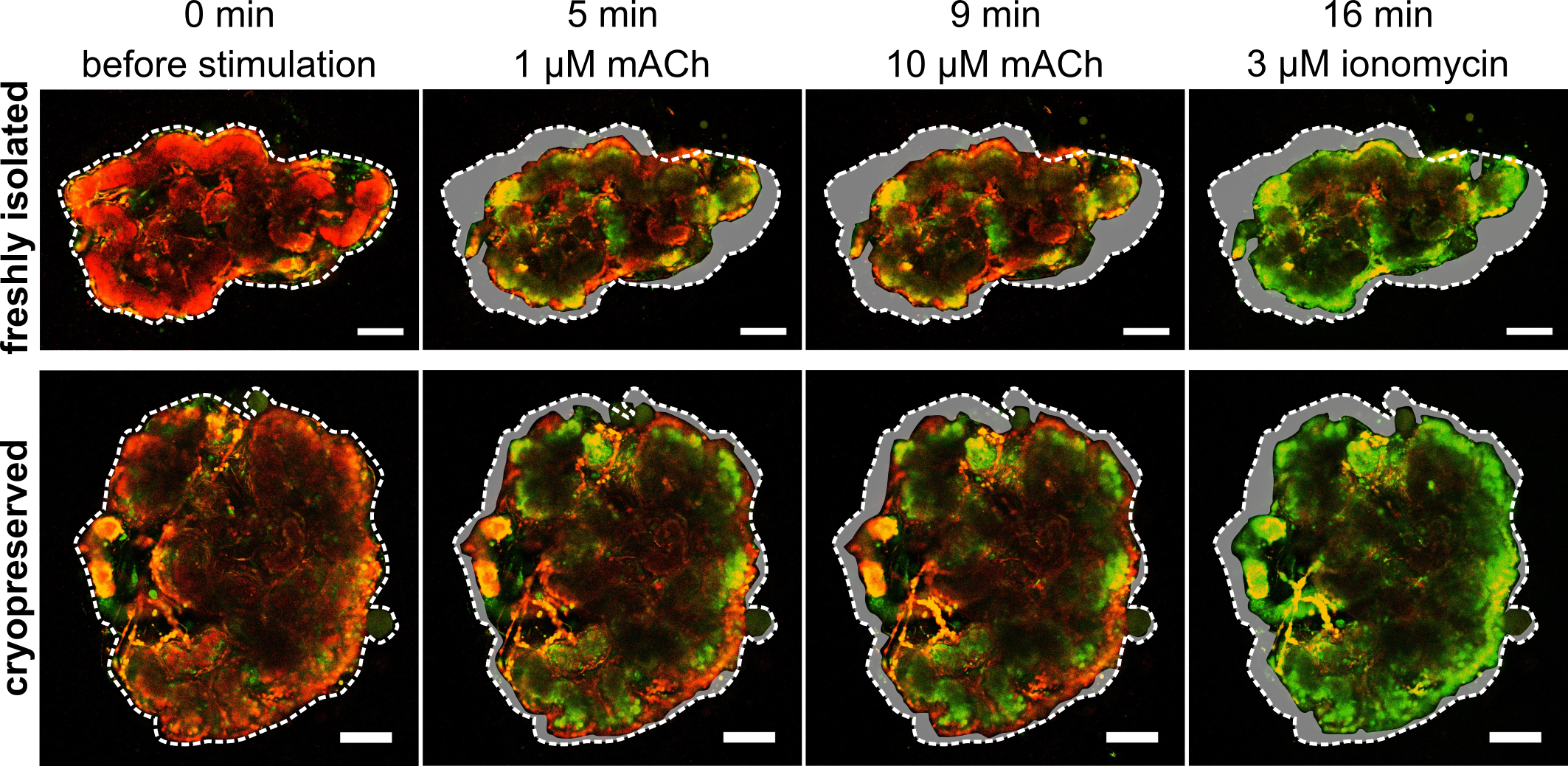

Supplement: S1 Fig — Freshly isolated and cryopreserved sweat glands differed in the intensity of contraction to cholinergic stimulus during calcium imaging. The freshly isolated sweat glands showed a greater contraction to 1 µ M mACh than the cryopreserved sweat glands. Although the contraction of the cryopreserved sweat glands was weaker than that of freshly isolated sweat glands, the contraction was clearly visible. Dotted line: outline of the sweat glands at the at the beginning of the experiment. Area highlighted in light grey: difference between the outline of the sweat gland before contraction at the time shown in the maximum projection. Acetyl-β-methylcholine (mACh), scale bars: 100 μm. (TIF) [file pone.0323255.s001.tif]

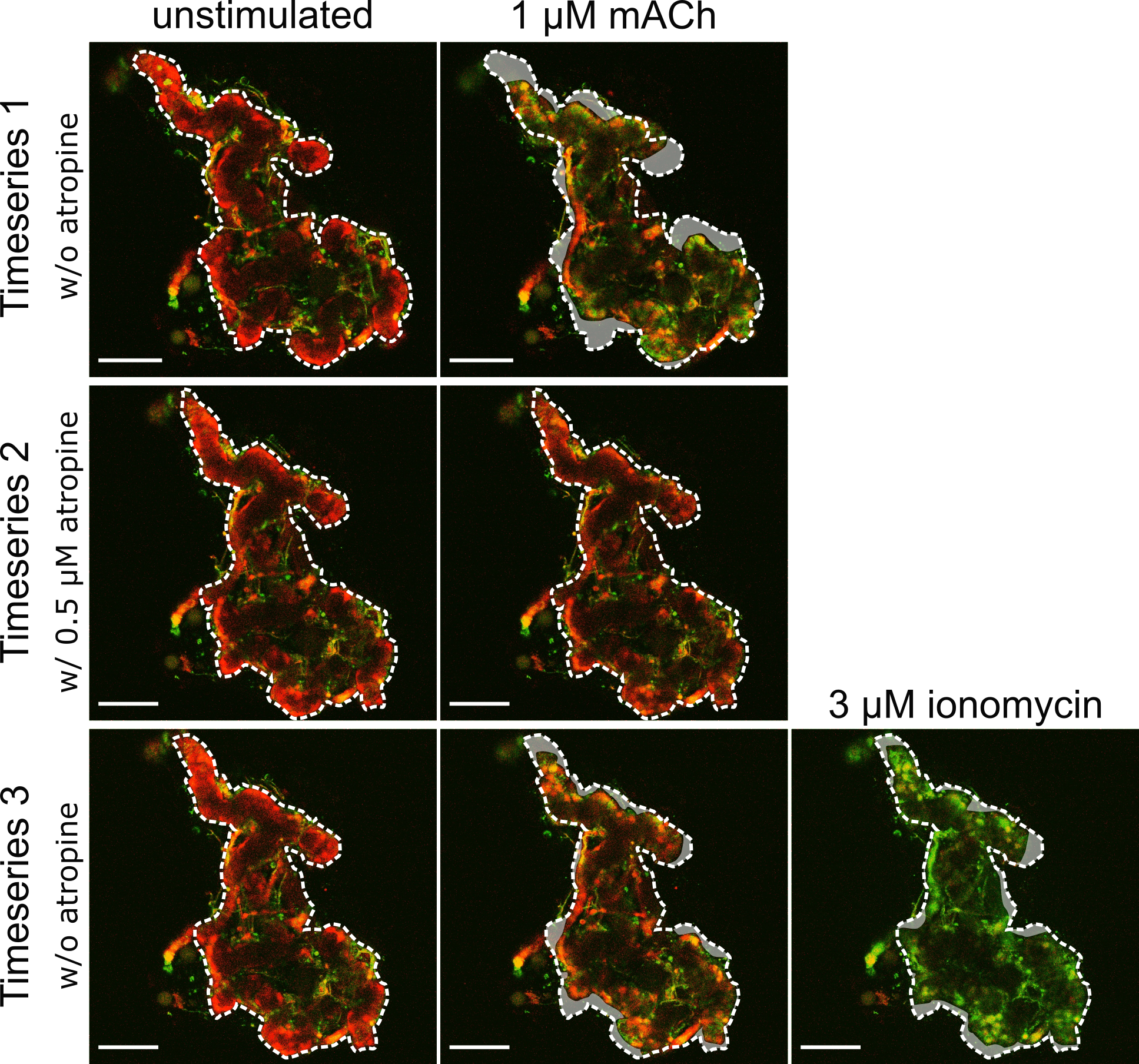

Supplement: S2 Fig — Representative images of a cholinergically stimulated rat sweat gland at different time points of the experiment. Timeseries 1 without atropine inhibition shows a clear and strong contraction of the secretory coil as well as a calcium response indicated by the colour change from Fura Red™ (red) to Fluo-4 (green). After mACh washout and application of atropine during the regeneration phase, the sweat gland is relaxed at the beginning and restimulation fails during ongoing atropine inhibition in timeseries 2. No contraction or colour change is seen. This indicates an effictive competitive inhibition of cholinergic calcium signalling by atropine. Atropine was then washed out and timeseries 3 followed the regeneration phase. Once again, the sweat gland was stimulated by cholinergic stimulation and showed a clear contraction and change in colour. This response was less pronounced than in timeseries 1. However, it clearly demonstrates the reversibility of atropine inhibition. The technical control of ionomycin results in a maximum colour change independent of physiological stimulation (positive control). Dotted line: outline of the sweat glands at the at the beginning of the experiment. Area highlighted in light grey: difference between the outline of the sweat gland before contraction at the time shown in the maximum projection. Acetyl-β-methylcholine (mACh), scale bars: 100 μm. (TIF) [file pone.0323255.s002.tif]
